# Investigation of the pharmacological mechanisms of Denglao Qingguan decoction in inhibiting viral pneumonia through network pharmacology and *in vitro* laboratory validation

**DOI:** 10.3389/fmed.2025.1708952

**Published:** 2026-01-12

**Authors:** Biao Lei, Shun Wang, Xuanxuan Li, Fang Chen, Wencong Lu, Bin Liu, Xiannan Chen, Ruihan Chen, Zhanyu Cui, Ai Li, Xi Ren, Linzhong Yu, Qinhai Ma

**Affiliations:** 1 Hospital of Integrated Traditional Chinese and Western Medicine, Southern Medical University, Guangzhou, Guangdong, China; 2 State Key Laboratory of Respiratory Disease, National Clinical Research Center for Respiratory Disease, Guangzhou Institute of Respiratory Health, The First Affiliated Hospital of Guangzhou Medical University, Guangzhou Medical University, Guangzhou, Guangdong, China; 3 School of Traditional Chinese Medicine, Shanghai University of Traditional Chinese Medicine, Shanghai, China; 4 Guangdong Women and Children Hospital, Guangzhou, Guangdong, China; 5 Baiyun District Maternal and Child Health Hospital, Guangzhou, Guangdong, China; 6 Third Level Research Laboratory of State Administration of Traditional Chinese Medicine, School of Traditional Chinese Medicine, Southern Medical University, Guangzhou, Guangdong, China; 7 Guangdong Provincial Key Laboratory of Chinese Medicine Pharmaceutics, School of Traditional Chinese Medicine, Southern Medical University, Guangzhou, Guangdong, China

**Keywords:** Denglao Qingguan decoction, inflammatory response, influenza virus, respiratory syncytial virus, severe acute respiratory syndrome coronavirus 2, traditional Chinese medicine

## Abstract

**Introduction:**

Respiratory viral infection poses a serious threat to human health, underscoring the need for effective agents to prevent and treat these conditions.

**Objective:**

This study aimed to investigate Denglao Qingguan decoction (DLQGD), a traditional formula used in the management of respiratory infections, and to elucidate its efficacy and mechanisms against viral pneumonia.

**Methods:**

Network pharmacology was employed to investigate the potential mechanism of DLQGD against viral pneumonia. The components of DLQGD were analyzed by High-Performance Liquid Chromatography-Quadrupole Time-of-Flight Mass Spectrometry (HPLC-Q-TOF MS). The antiviral effects of DLQGD against respiratory syncytial virus (RSV), influenza virus and severe acute respiratory syndrome coronavirus 2 (SARS-CoV-2) were determined by cytopathic effect or MTT assays. The inhibitory effects of DLQGD on RSV or SARS-CoV-2-induced inflammatory response were determined by reverse transcriptase-quantitative PCR.

**Results:**

A total of 66 active ingredients were identified in DLQGD. Network pharmacology showed that DLQGD could regulate a total of 122 targets involved in viral pneumonia. The 50% inhibitory concentration (IC_50_) of DLQGD against RSV, SARS-CoV-2, and H3N2 was 0.4824, 1.16, and 1.592 mg/mL. The selectivity index (SI) of DLQGD against RSV, SARS-CoV-2, and H3N2 was 15.82, 5.96, and 5.74, respectively. Furthermore, DLQGD significantly inhibit the viral titer of cell culture supernatants during SARS-CoV-2 infection. DLQGD markedly reduced the mRNA expression of IL6, TNF, CXCL8, and CXCL10 in Huh-7 cells infected by SARS-CoV-2. In addition, DLQGD decreased the mRNA expression of IL6, TNF, IL1B, CXCL8, CXCL10, and CCL5 in HEp-2 cells infected by RSV. DLQGD could downregulate the protein expression of phosphorylated Stat3, Akt, and Erk1/2.

**Conclusion:**

Collectively, these findings indicate that DLQGD exhibits antiviral and anti-inflammatory activities, suggesting that it could be developed into a therapeutic constituent for respiratory viral infections.

## 1 Introduction

Lower respiratory tract infections cause a serious burden and high mortality worldwide (1). Respiratory syncytial virus (RSV), influenza virus and severe acute respiratory syndrome coronavirus 2 (SARS-CoV-2) are common pathogens resulting in respiratory tract infection or viral pneumonia (2). RSV can cause acute lower respiratory infection (ALRI) and high mortality among children and the elderly population (aged > 60 years) (3). Annually, it can cause approximately 33 million instances of ALRI among children under the age of 5, and lead to 3.6 million hospitalizations and 118,200 fatalities (3, 4). SARS-CoV-2 can cause a global pandemic and has led to more than 7 million deaths in recent years. Influenza virus infection can cause up to 500 million severe cases and 600 thousand deaths annually (5). Upon sensing these respiratory viruses by pattern recognition receptors (PRRs), inflammatory signaling pathways would be activated. High levels of cytokines and chemokines would be released during this process, accompanied by massive inflammatory cell infiltration in the sites of infection. The recruitment of inflammatory cells such as neutrophils and macrophages, would destroy normal lung tissues or even result in acute respiratory distress syndrome (ARDS). Controlling virus-induced inflammatory response is an important treatment strategy in respiratory infectious diseases (6). It is necessary to control the overactivated inflammatory response induced by respiratory virus infection. Antiviral drugs can effectively inhibit virus proliferation. However, they could not inhibit the activation of inflammatory cells and signaling pathways to control the overactivated inflammatory response. Corticosteroids are potent anti-inflammatory drugs. However, corticosteroids play a controversial role in managing severe viral pneumonia, encompassing cases caused by the influenza virus, SARS-CoV-2, RSV, and other respiratory viruses (7–9). Corticosteroid therapy could complicate bacterial and fungal infections during influenza (10). More strategies are required to modulate the excessive inflammatory response associated with respiratory viral infections.

Traditional Chinese Medicines (TCMs) have been widely used for the treatment of RSV infection, influenza and COVID-19 in China (11, 12). TCMs can accelerate the recovery of symptoms, inhibit viral replication and alleviate the formation of cytokine storm during respiratory virus infection (11, 13). Denglao Qingguan decoction (DLQGD) consists of 14 herbal medicines, including *Flos Lonicerae* (Jinyinhua), *Flo Chrysanthem* (Juhua), *Morus alba* L. (Sangye), *Herba Taraxaci* (Pugongying), *Herba Menthae* (Bohe), *Pogostemon Cablin (Blanco) Benth* (Guanghuoxiang), *Radix Glycyrrhizae* (Gancao), *Semen Armeniacae Amarum* (Xingren), *Semen Juglandis* (Taoren), *Radix Platycodonis* (Jiegeng), *Rhizoma Imperatae* (Baimaogen), *Pericarpium Citri Reticulatae* (Jupi), and *Fructus Hordei Germinatus* (Maiya), and *Radix Fici Simplicissimae* (Wuzhimaotao). DLQGD can not only clear heat and remove toxins, but also disperse the lung and transform dampness. It has been employed to prevent and treat respiratory infections. A previous study indicated that DLQGD exhibits antiviral effects against HCoV-229E and can inhibit the inflammatory response induced by HCoV-229E (14). Multiple herbal medicines possess antiviral and anti-inflammatory effects (15–17). The components of *Flos Lonicerae*, *Pogostemon Cablin (Blanco) Benth*, and *Radix Glycyrrhizae* can inhibit the replication of influenza virus (15, 18, 19). The ingredients of *Radix Platycodonis* and *Flos Lonicerae* can alleviate RSV-induced inflammation (20, 21). The components of *Radix Glycyrrhizae* exhibit protective effects against SARS-CoV-2 infection (22, 23). DLQGD may be a promising agent for the treatment of respiratory virus infection. But the effects and mechanisms remain unclear. Unlike most conventional agents that target single molecules, TCMs consist of various components that interact with numerous biological molecules and multiple signaling pathways. Network pharmacology can integrate systems biology and molecular pharmacology to study interactions between components and targets by establishing clarity networks (24, 25). It has become a powerful strategy for uncovering the complex mechanisms of TCMs in disease treatment.

In this study, High-Performance Liquid Chromatography-Quadrupole Time-of-Flight Mass Spectrometry (HPLC-Q-TOF MS) was utilized to characterize the constituents of DLQGD. Network pharmacology was employed to investigate the mechanisms of DLQGD in the treatment of viral pneumonia and its protective effects on respiratory virus infection were evaluated *in vitro*. Our findings indicated that DLQGD exhibited antiviral and anti-inflammatory effects against respiratory virus infection.

## 2 Materials and methods

### 2.1 Screening of the targets of DLQGD

The SMILES of active ingredients were collected in PubChem website.^1^ The SMILES of compounds were used to collect related targets of the ingredients from the SwissTargetPrediction database.^2^ Gene names that correspond to these targets were obtained from the protein database Uniprot.^3^

### 2.2 Collection of therapeutic targets involved in viral pneumonia

Targets involved in viral pneumonia were collected from Genecard and OMIM databases. The overlapping targets between DLQGD and related targets involved in viral pneumonia were obtained from an online Venn diagram drawing tool, Venny version. 2.1.^4^

### 2.3 Protein–protein interaction network construction

Therapeutic targets associated with viral pneumonia were analyzed by STRING database,^5^ with the species parameter restricted to “Homo sapiens” and a confidence score threshold set at ≥ 0.7. The results were visualized and analyzed by Cytoscape 3.7.2.

### 2.4 Gene ontology and Kyotoencyclopedia of genes and genomes pathway enrichment analysis

Therapeutic targets of DLQGD related to viral pneumonia were submitted to Database for Annotation, Visualization, and Integrated Discovery (DAVID) web server^6^ for GO biological processes and KEGG pathway enrichment analysis with FDR < 0.05 and *P* < 0.05 as cut-off values.

### 2.5 Network construction

To analyze the interactions between ingredients and therapeutic targets, a “Drug-Compound-Target-Disease” network was constructed using Cytoscape 3.7.2. Within this network, nodes represent herbs, compounds, and targets, while edges depict the relationships among them. The importance of each node was evaluated based on its “degree,” defined as the number of connections linked to the node, which serves as an indicator of its topological significance. The overall network properties were analyzed using the “Network Analyzer” function.

### 2.6 Molecular docking

To identify the key bioactive compounds responsible for the pharmacological effects, we assessed the binding sites and binding affinities between these compounds and the core targets of DLQGD using the CB-Dock web server.^7^ The selection of compounds was based on their relevance as determined through network analysis conducted with Cytoscape (version 3.7.2). The three-dimensional structure of each protein in PDB format was retrieved from the Protein Data Bank database, while the 3D structures of the key ligands (in.sdf format) were obtained from PubChem. The binding affinities were evaluated based on Vina scores and cavity dimensions, both provided by CB-Dock. Molecular visualization of the binding sites was performed using PyMOL software.

### 2.7 Reagents

DLQGD was provided by Guangdong Denglao herbal tea Pharmaceutical Group (Guangzhou, China) and prepared as previously mentioned (14), which is composed of 15 g *Flos Lonicerae* (Jinyinhua), 15 g *Flo Chrysanthem* (Juhua), 15 g *Morus alba* L. (Sangye), 15 g *Herba Taraxaci* (Pugongying), 6 g *Herba Menthae* (Bohe), 15 g *Pogostemon Cablin (Blanco) Benth* (Guanghuoxiang), 6 g *Radix Glycyrrhizae* (Gancao), 15 g *Semen Armeniacae Amarum* (Xingren), 15 g *Semen Juglandis* (Taoren), 15 g *Radix Platycodonis* (Jiegeng), 30 g *Rhizoma Imperatae* (Baimaogen), 6 g *Pericarpium Citri Reticulatae* (Jupi), and 30 g *Fructus Hordei Germinatus* (Maiya), and 30 g *Radix Fici Simplicissimae* (Wuzhimaotao). After weighing 14 herbs of DLQGD proportionally, DLQGD (198 g) was soaked in 1980 mL water for 30 min and were boiled twice for 1.5 h each time. Next, all of the decoctions were mixed and concentrated, which were then stored at -80°C for 24 h. Finally, the freeze-dried powder was obtained using a lyophilizer (Christ, Germany).

### 2.8 Identification of active compounds of DLQGD using HPLC-Q-TOF MS

DLQGD (2 g) was prepared in 20% methanol and mixed for 1 min. The volume of samples was adjusted to 10 mL and the samples were then centrifuged for 10 min at 12,000 rpm. The supernatants were analyzed by HPLC-Q-TOF MS. The chromatographic conditions were set as follows: The Poroshell SB-Aq column (150 mm × 3.0 mm, 2.7 μm) was used at a flow rate of 0.4 mL/min and a volume of 5 μL. The mobile phase consisted of 0.1% formic acid in water (A) and methanol (B). The multi-step linear elution gradient program was as follows: 0–6 min, 0% B; 6∼20.0 min, 0%∼10% B; 20.0∼35.0 min, 10%∼15% B; 35.0∼50.0 min, 15%∼25% B; 50.0∼80.0 min, 25%∼32% B; 80.0∼95.0 min, 32%∼42% B; 95.0∼115.0 min, 42%∼65% B; 115.0∼120.0 min, 65%∼80% B; 120.0∼125.0 min, 80% B; 125.1∼130.0 min, 0% B. The mass spectrometer was conducted as follow: Agilent Dual AJS ESI ion source, positive and negative ion scanning; drying gas (N2), 300°C; nebulizer gas (N2), 35 psi; drying gas (N2), 8 L/min; sheath gas, 350°C; sheath gas, 11 L/min; electrospray voltage, 3,500 V; capillary exit voltage, 150 V; cone voltage, 65 V; octupole voltage, 750 V; scan range: 100–1,000 m/z; collision energy (10, 20, 40 eV).

### 2.9 Cells and viruses

Human Epithelioma-2 (HEp-2), Madin-Darby canine kidney (MDCK), Vero E6 and human hepatocellular carcinoma (Huh-7) cell lines were prepared as previously mentioned (11, 26). HEp-2, Vero E6 and Huh-7 cells were cultured in Dulbecco’s modified Eagle’s medium (DMEM, Gibco, United States) with 10% fetal bovine serum (FBS) and 1% penicillin-streptomycin (Gibco, United States) in a 5% CO_2_ incubator at 37 °C. MDCK cells were cultured in DMEM/F12 (1:1) medium (Gibco, United States) with 10% FBS. The medium used for the cytotoxic and antiviral assays related to SARS-CoV-2 or RSV contained 2% of serum. RSV Long strain A was purchased from Guangzhou GeneBank Biotechnology Co., Ltd. SARS-CoV-2 (Genebank accession no. MT123290.1) was a clinical strain isolated from the First Affiliated Hospital of Guangzhou Medical University. A/Aichi/68 (H3N2) was purchased from the Centers for Disease Control and Prevention (CDC). The viral titer was determined by the Reed–Muench method as previously mentioned (26).

### 2.10 Cell viability assay

HEp-2, MDCK, Huh-7 and Vero E6 cells (5 × 10^4^ cells/well) were inoculated in 96-well plates. After 24 h of incubation, cells were treated with two-fold serial dilutions of DLQGD for 72 h, then washed twice and stained with 0.5 mg/mL MTT solution at 37 °C. Following staining for 4 h, the solution was removed, and 100 μL of DMSO per well was added to dissolve the formed formazan crystals. The absorbance in each well at 490 nm was examined using a Multiskan Spectrum Reader (Thermo Fisher, United States). The 50% toxicity concentration (TC_50_) value of DLQGD to HEp-2, MDCK, Huh-7 and Vero E6 cells was calculated using GraphPad Prism 8.0.

### 2.11 Cytopathic effect inhibition assay

HEp-2 cells, at a density of 5 × 10^4^ cells per well, were seeded into a 96-well plate. The HEp-2 monolayers were washed twice with PBS. Two-fold serial dilutions of DLQGD were applied to the cells, accompanied by 100 TCID_50_ of RSV. After incubation for 72 h, 100 μL DMEM was replenished to each well. CPE was observed after incubating for 96 h. The 50% inhibitory concentration (IC_50_) was determined using the Reed–Muench method.

Similarly, a Vero E6 cell monolayer was washed twice with PBS and then inoculated with 100 TCID_50_ of SARS-CoV-2 for 2 h. This was followed by incubation with varying concentrations of DLQGD. After incubation for 72 h, Vero E6 cells were examined microscopically to record CPE. IC_50_ of DLQGD was calculated by the Reed–Muench method.

A MDCK cell monolayer was washed twice with PBS and subsequently inoculated with 100 TCID_50_ of H3N2 for 2 h, followed by incubation with different doses of DLQGD. After incubation for 48 h, MDCK cells were microscopically examined for CPE. IC_50_ of DLQGD was calculated by the Reed–Muench method.

### 2.12 RNA isolation and reverse transcriptase-quantitative PCR analysis

The HEp-2 monolayers cultured in a 12-well plate, were washed twice with PBS. Two-fold serially dilutions of DLQGD were added to the cells accompanied by 100 TCID_50_ of RSV. A Huh-7 cell monolayer cultured in a 12-well plate, was washed twice with PBS and subsequently inoculated with 100 TCID_50_ of SARS-CoV-2 for 2 h, followed by incubation with different doses of DLQGD. After 48 h, total RNA was harvested by TRIzol (Invitrogen, MA, United States) in accordance with the specification, then reverse transcribed via the PrimeScript™ RT Master Mix kit (Takara Bio, Japan). RT-PCR was conducted on reverse transcription samples using SYBR Premix Ex Tap™ II (Takara Bio, Japan). Primer sequence of mRNA for RT-qPCR was laid out in Table 1. PCR results were read with ABI PRISM^®^ 7500 Real-time PCR detection system (Applied Biosystems Co., United States). The relative expression level of mRNA was analyzed.

**TABLE 1:** Primer sequence for RT-qPCR.

Target gene	Direction	Sequence (5′–3′)
IL6	Forward	CGGGAACGAAAGAGAAGCTCTA
	Reverse	CGCTTGTGGAGAAGGAGTTCA
CXCL8	Forward	TTGGCAGCCTTCCTGATTTC
	Reverse	TATGCACTGACATCTAAGTTCTTTAGCA
CXCL10	Forward	GAAATTATTCCTGCAAGCCAATTT
	Reverse	TCACCCTTCTTTTTCAT-TGTAGCA
TNF	Forward	AACATCCAACCTTCCCAAACG
	Reverse	GACCCTAAGCCCCCAATTCTC
CCL5	Forward	CAGCAGTCGTCTTTGTCACC
	Reverse	GTTGATGTACTCCCGAACCC
IL1B	Forward	GCACGATGCACCTGTACGAT
	Reverse	AGACATCACCAAGCTTTTTTGCT
GAPDH	Forward	GAAGGTGAAGGTCGGAGTC
	Reverse	GAAGATGGTGATGGGATTTC

### 2.13 Western blot

Western blots were performed as previously mentioned (11). The primary antibodies including anti-Stat3 antibody (Stat3, cat# 4904S, CST, United States), anti-phospho-Stat3 antibody (p-Stat3, cat# 9145, CST, United States), anti-Akt antibody (Akt, cat# 4685, CST, United States), anti-phospho-Akt antibody (p-Akt, cat# 9217, CST, United States), anti-phospho-p44/42 MAPK antibody (p-Erk1/2, cat# 4370, CST, United States), anti-p44/42 MAPK antibody (Erk1/2, cat# 9120, CST, United States) or anti-β-Actin antibody (cat# 8457, CST, United States) were incubated overnight at 4°C, followed by the incubation with corresponding secondary antibodies (anti-rabbit IgG, cat# SA00001-2, proteintech, United States or anti-mouse IgG cat# SA00001-1, proteintech, United States) for 1 h.

### 2.14 Statistical analysis

Statistical analysis was performed using Prism GraphPad 8 software. Multiple group comparisons were conducted using either Bonferroni or Dunnett’s test, depending on the outcome of homogeneity of variance testing. Significance levels were indicated as follows: *, *p* < 0.05; **, *p* < 0.01 or ***, *p* < 0.001.

## 3 Results

### 3.1 Identification of the components of DLQGD

**FIGURE 1:**
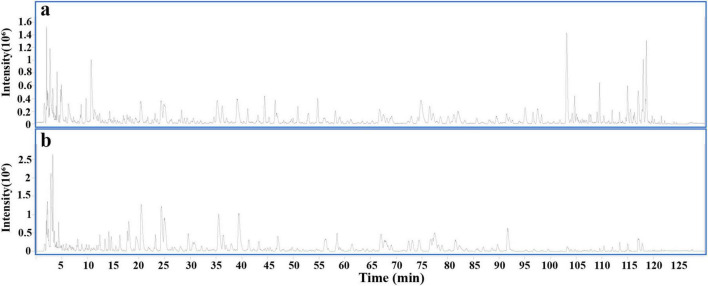
Total ion chromatogram of DLQGD by HPLC-Q-TOF MS. **(a)** Positive ion modes. **(b)** Negative ion modes.

To explore the components of DLQGD, HPLC-Q-TOF MS analysis was employed to identify the chemical composition profiling. Overall, a total of 66 compounds were identified in DLQGD (Table 2 and Figure 1).

**TABLE 2:** The ingredients of DLQGD.

Number	t/min	Name	Molecular formula	CAS
D1	2.03	Gluconic acid	C6H12O7	526-95-4
D2	2.14	Proline	C5H9NO2	147-85-3
D3	2.15	Quinic acid	C7H12O6	77-95-2
D4	2.15	Sucrose	C12H22O11	57-50-1
D5	2.38	Betaine	C5H11NO2	107-43-7
D6	2.38	Malic acid	C4H6O5	6915-15-7
D7	2.84	Trigonelline	C7H7NO2	6138-41-6
D8	2.84	Stachydrine	C7H13NO2	1195-94-4
D9	2.85	Citric acid	C6H8O7	77-92-9
D10	3.31	L-Pyroglutamic acid	C5H7NO3	98-79-3
D11	4.13	Phenylalanine	C9H11NO2	63-91-2
D12	4.79	Adenine	C5H5N5	73-24-5
D13	6.28	Hordenine	C10H15NO	539-15-1
D14	9.72	Guanosine	C10H13N5O5	118-00-3
D15	10.77	Adenosine	C10H13N5O4	58-61-7
D16	11.71	Protocatechuic acid	C7H6O4	99-50-3
D17	14.16	Xanthohumol	C21H22O5	56754-58-1
D18	14.58	Mandelic acid-β-gentiobioside or isomer	C20H28O13	–
D19	16.26	3,4-Dihydroxybenzaldehyde	C7H6O3	139-85-5
D20	17.77	isorhamnetin	C16H12O7	480-19-3
D21	18.13	5-Hydroxyferulic acid	C10H10O5	1782-55-4
D22	19.53	Caftaric acid	C13H12O9	67879-58-7
D23	20.34	Neochlorogenic acid	C16H18O9	906-33-2
D24	21.86	Prunasin	C14H17NO6	99-18-3
D25	23.13	Benzyl-β-gentiobioside	C19H28O11	–
D26	24.30	L-amygdalin	C20H27NO11	29883-16-7
D27	24.88	D-amygdalin	C20H27NO11	29883-15-6
D28	29.32	Monotropein	C16H22O11	–
D29	30.14	Caffeic acid	C9H8O4	331-39-5
D30	30.72	Secologanic acid	C16H22O10	–
D31	35.50	Cryptochlorogenic acid	C16H18O9	905-99-7
D32	36.20	Sweroside	C16H22O9	14215-86-2
D33	39.46	Chlorogenic acid	C16H18O9	327-97-9
D34	43.31	Verbenalin	C18H28O9	–
D35	46.81	Secoxyloganin	C17H24O11	58822-47-2
D36	61.16	Azelaic acid	C9H16O4	123-99-9
D37	66.75	Liquiritin	C21H22O9	551-15-5
D38	67.45	Acacetin	C16H12O5	480-44-4
D39	67.69	Cichoric acid	C22H18O12	70831-56-0
D40	68.16	Naringin	C27H32O14	10236-47-2
D41	68.85	Isolicoflavonol	C20H18O6	94805-83-1
D42	72.12	Acteoside	C29H36O15	61276-17-3
D43	72.94	Liguiritigenin-7-O-D-apiosyl-4’-O-D-Glucoside	C26H30O13	19979-12-8
D44	74.10	Naringenin	C15H12O5	480-41-1
D45	74.68	Psoralen	C11H6O3	66-97-7
D46	76.43	Hesperidin	C28H34O15	520-26-3
D47	76.78	Isoacteoside	C29H36O15	61303-13-7
D48	77.02	Quercetin	C15H10O7	117-39-5
D49	77.02	Isochlorogenic acid B	C25H24O12	14534-61-3
D50	77.02	Quercetin-7-o-β-D-glucopyranoside	C21H20O12	491-50-9
D51	77.72	Rosmarinic acid	C18H16O8	20283-92-5
D52	79.93	Formononetin glucoside	C22H22O9	486-62-4
D53	81.10	Isochlorogenic acid A	C25H24O12	2450-53-5
D54	81.68	Luteolin-7-O-β-D-glucoside	C21H20O11	5373-11-5
D55	89.38	Isoliquiritin	C21H22O9	5041-81-6
D56	91.36	Isochlorogenic acid C	C25H24O12	57378-72-0
D57	96.49	Neodiosmin	C28H32O15	–
D58	97.31	Bergapten	C12H8O4	484-20-8
D59	102.91	Buddleoside	C28H32O14	480-36-4
D60	103.26	Luteolin	C15H10O6	491-70-3
D61	114.92	24-Hydroxyl-glycyrrhizin or isomer	C42H62O17	–
D62	115.38	Sinensetin	C20H20O7	2306-27-6
D63	116.90	Glycyrrhizic acid	C42H62O16	1405-86-3
D64	117.72	Nobiletin	C21H22O8	478-01-3
D65	118.42	Tangeretin	C20H20O7	481-53-8
D66	118.42	3,3′,4′,5,6,7,8-heptamethoxyflavone	C22H24O9	1178-24-1

### 3.2 Potential targets of DLQGD for treating viral pneumonia

**FIGURE 2:**
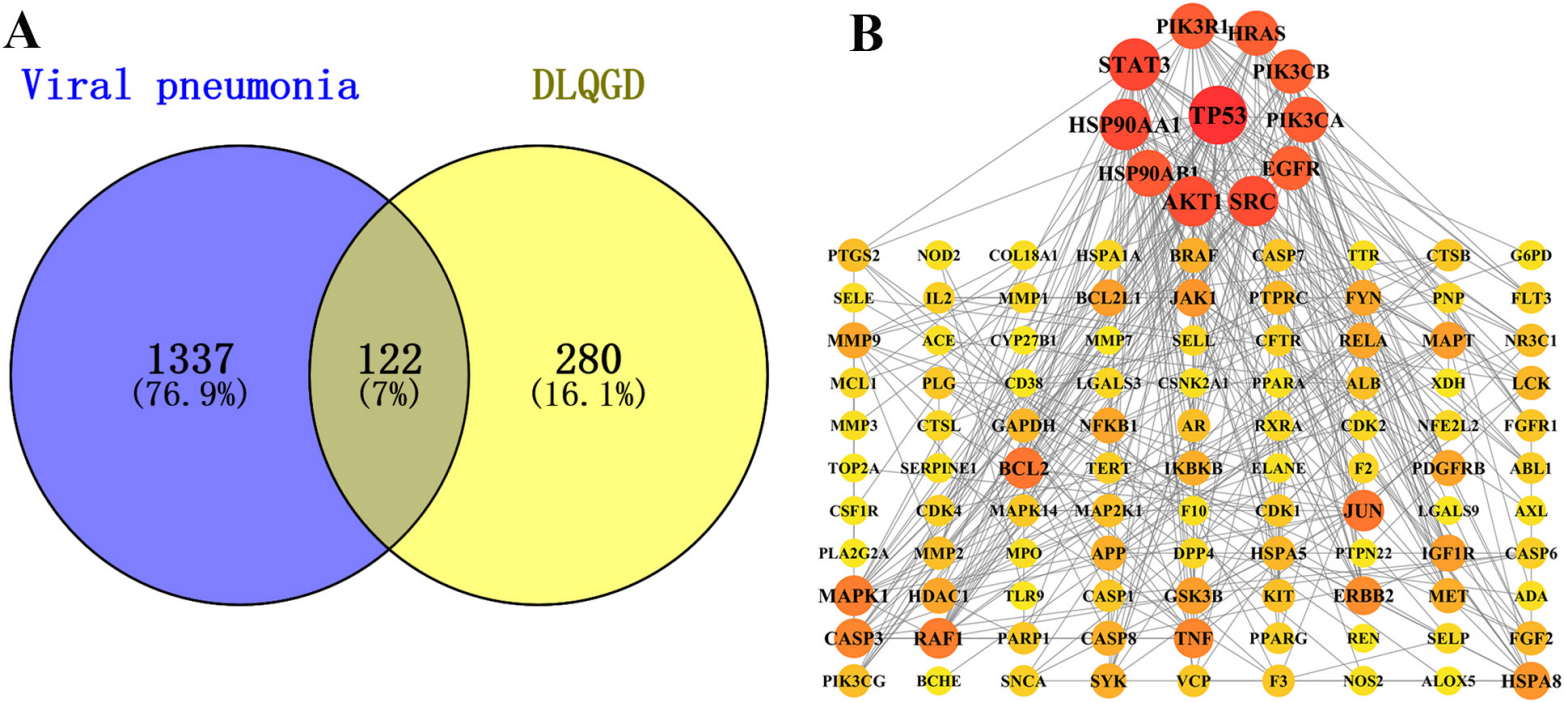
The potential targets of DLQGD for the treatment of viral pneumonia. **(A)** The Venn diagram of targets between targets of DLQGD and targets involved in viral pneumonia. The overlapping shape represented 122 viral pneumonia-related targets regulated by DLQGD. **(B)** The protein–protein interactions of 122 targets. The darker and larger circles represent the more important targets regulated by DLQGD.

According to the data of network pharmacology, a total of 1459 targets involved in viral pneumonia and 402 DLQGD-related targets were collected. A total of 122 targets might account for the therapeutic effects of DLQGD in the treatment of viral pneumonia (Figure 2A). TP53, STAT3, HSP90AA1, HSP90AB1, AKT1, SRC, EGFR, PIK3CA, PIK3CB, HRAS, and PIK3R1 were key genes regulated by DLQGD (Figure 2B).

### 3.3 GO and KEGG enrichment analysis

**FIGURE 3:**
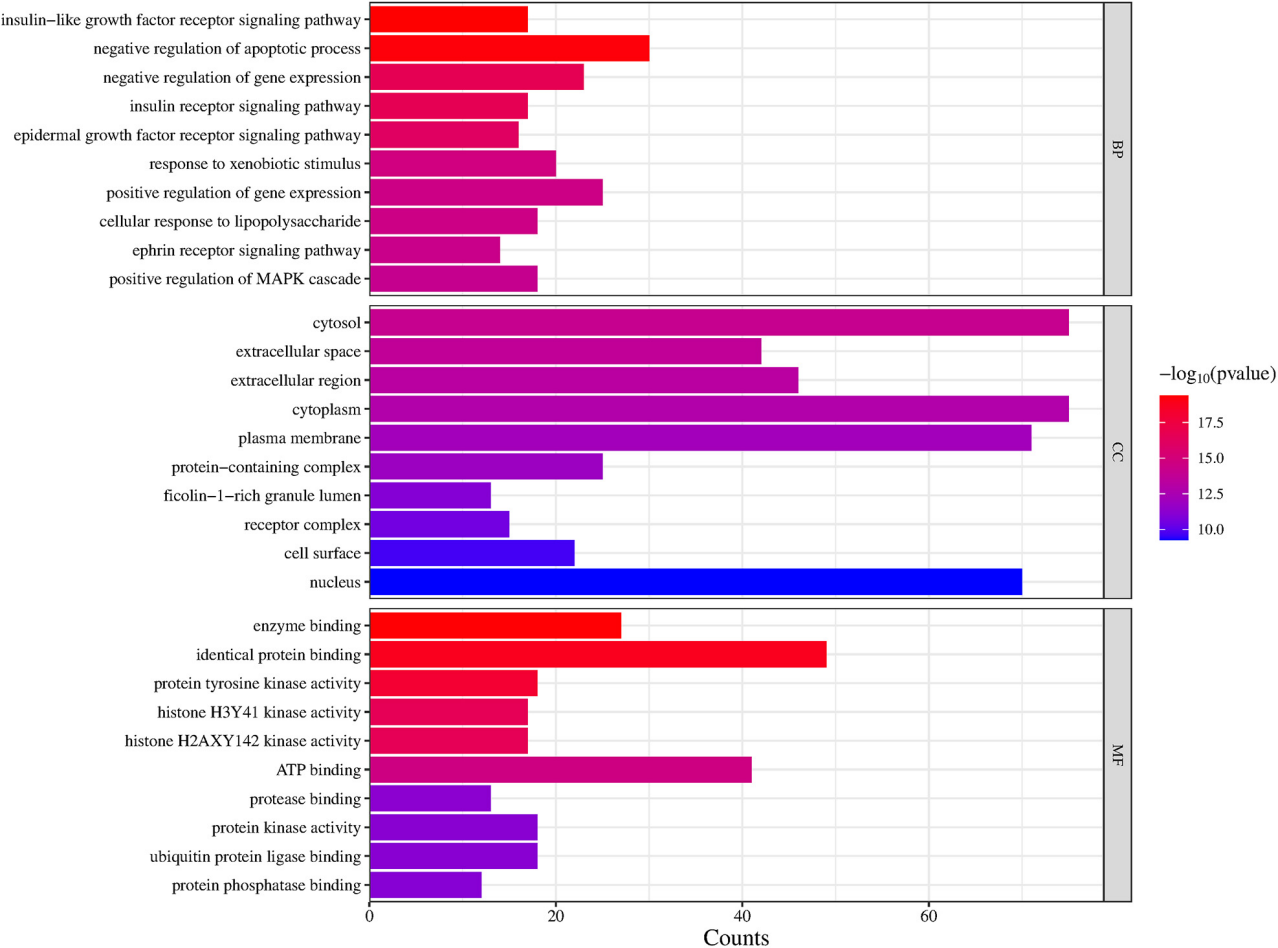
Top 10 items of GO enrichment analysis regulated by DLQGD.

To explore the potential functional pathways regulated by DLQGD, GO and KEGG enrichment analysis were carried out on 122 identified targets. A total of 776 items were identified by GO enrichment analysis, including 536 biological process (BP) terms, 68 cellular component (CC) terms and 172 molecular function (MF) terms. The top 10 terms in BP, CC, and MF categories were shown in Figure 3. Among them, BP such as insulin-like growth factor receptor signaling pathway, negative regulation of apoptotic process, negative regulation of gene expression, insulin receptor signaling pathway and epidermal growth factor receptor signaling pathway, may contribute to the therapeutic effects of DLQGD (Figure 3). The results of MF showed that the mechanism of DLQGD in the treatment of viral pneumonia was mainly related to enzyme binding, identical protein binding, protein tyrosine kinase activity, histone H2AXY142 kinase activity and histone H3Y41 kinase activity (Figure 3).

**FIGURE 4:**
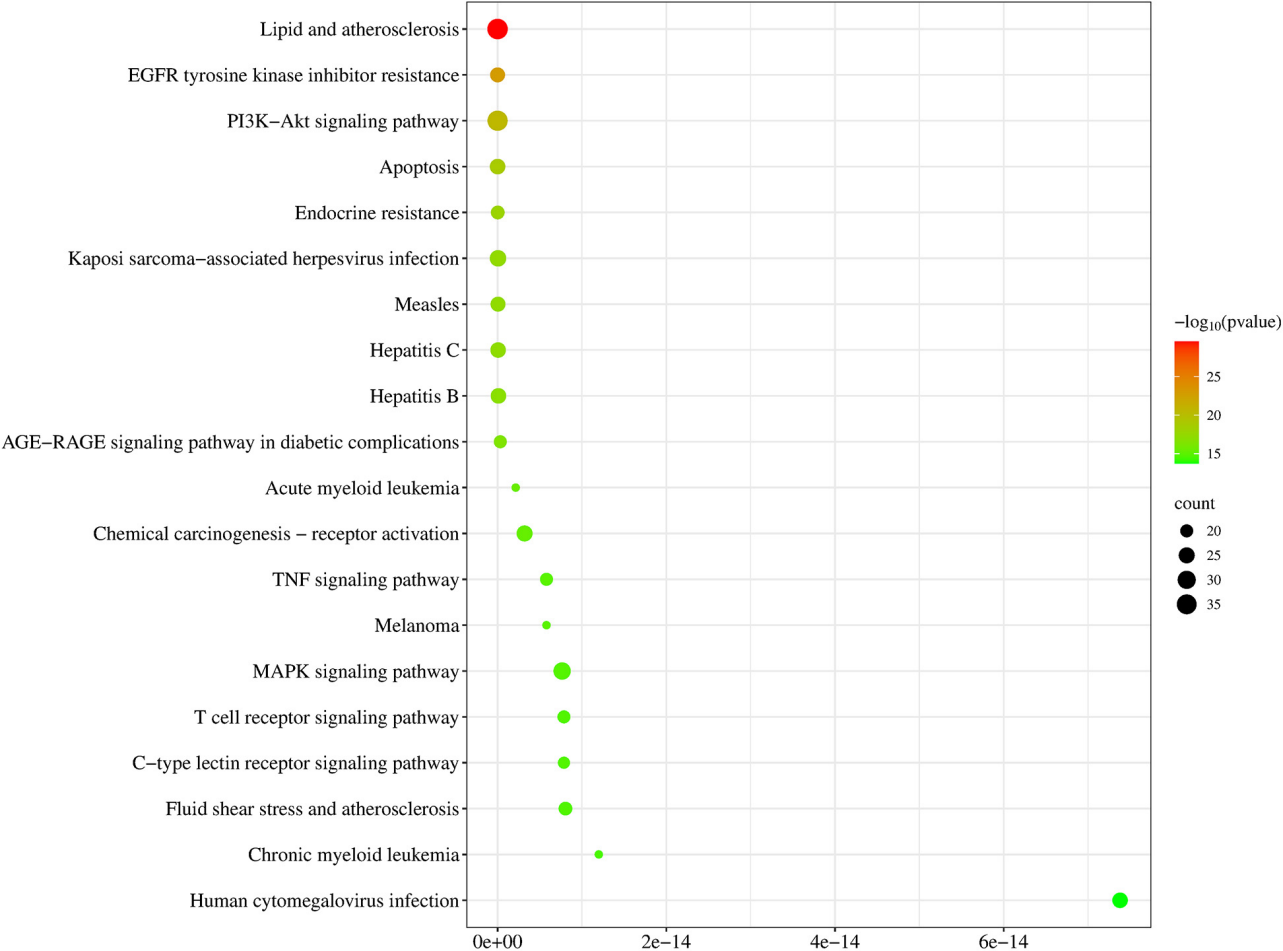
Top 20 items of KEGG enrichment analysis regulated by DLQGD.

A total of 776 items were identified by KEGG enrichment analysis and are shown in Figure 4. Among them, lipid and atherosclerosis, EGFR tyrosine kinase inhibitor resistance, PI3K-Akt signaling pathway, apoptosis and endocrine resistance, etc., were key items regulated by DLQGD. Among the top 20 items, PI3K-Akt signaling pathway, TNF signaling pathway, MAPK signaling pathway, etc. were related to the inflammatory response induced by the virus.

### 3.4 Target–active ingredient network

**FIGURE 5:**
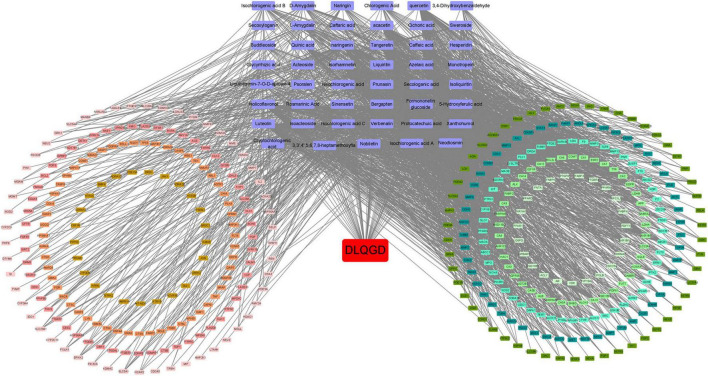
Target-active ingredient network. Purple nodes indicate the active compounds, while the red node represents DLQGD. The remaining nodes correspond to key targets. The edges illustrate the interactions between these compounds and targets.

Cytoscape software was used to construct the target-active ingredient network (Figure 5). As shown in Figure 5 and Supplementary Table 1, tangeretin, isorhamnetin, isolicoflavonol, luteolin, sinensetin, acacetin, and quercetin are key compounds that contribute to the therapeutic effects.

### 3.5 Molecular docking

**FIGURE 6:**
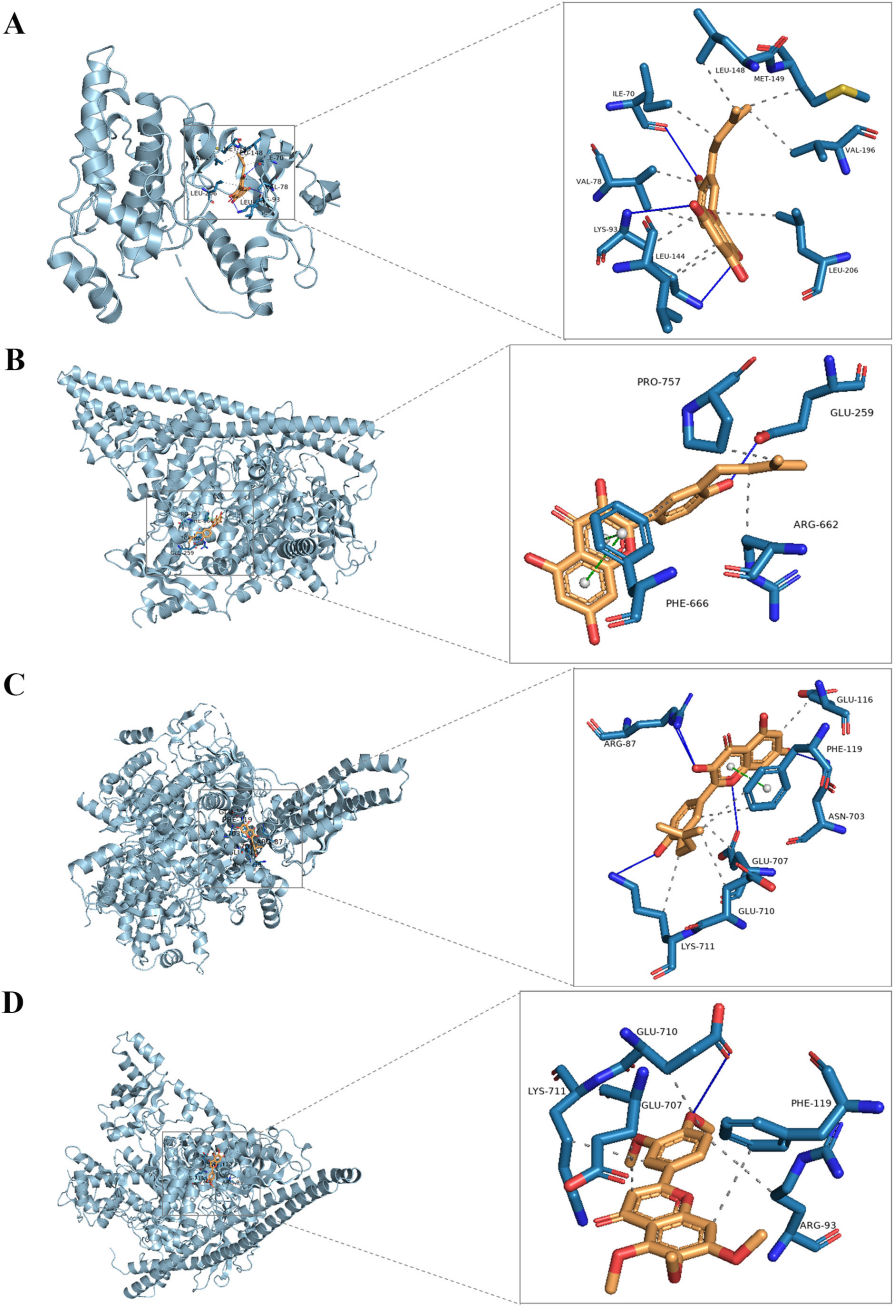
The representative examples of interactions of the key compounds with targets. **(A)** The interaction of JUN (gray) with isolicoflavonol (golden); **(B)** the interaction of PIK3CA (gray) with isolicoflavonol (golden); **(C)** the interaction of PIK3R1 (gray) with isolicoflavonol (golden); **(D)** the interaction of PIK3R1 (gray) with sinensetin (golden).

Molecular docking was employed to identify key targets and compounds potentially associated with the therapeutic effects of DLQGD in treating viral pneumonia. Key compounds were prioritized based on their network relevance, and critical targets involved in virus-induced inflammation were selected using the protein-protein interaction (PPI) network (Figure 2B). The Vina scores, which reflect binding affinity, indicate that lower values correspond to stronger and more stable interactions between the compounds and their receptors. As summarized in Table 3, tangeretin, isorhamnetin, isolicoflavonol, luteolin, sinensetin, and acacetin exhibited superior binding activities with PIK3CA, PIK3R1, and JUN. The representative examples of interactions between these targets and compounds are illustrated in Figure 6.

**TABLE 3:** The Vina scores of molecular docking.

Protein	STAT3 (6NJS)	AKT1 (8R5K)	PIK3CA (4JPS)	PIK3R1 (7PG6)	JUN (3OY1)	MAPK1 (2OJJ)
Tangeretin	−6.6	−5.9	−7.8	−8.9	−8.7	−7.7
Isorhamnetin	−8.1	−6.7	−8.6	−8.5	−8.3	−8
Isolicoflavonol	−7.7	−7.4	−9.1	−10.2	−9.1	−8.8
Luteolin	−7.8	−6.8	−8.7	−9	−9	−8
Sinensetin	−6.5	−5.8	−8.1	−9.2	−8.2	−7.5
Acacetin	−7.2	−6.7	−8.5	−S9.2	−8.6	−7.8
Quercetin	−8.1	−6.7	−8.4	−8.9	−8.9	−8

### 3.6 Antiviral effects of DLQGD against respiratory viruses *in vitro*

**FIGURE 7:**
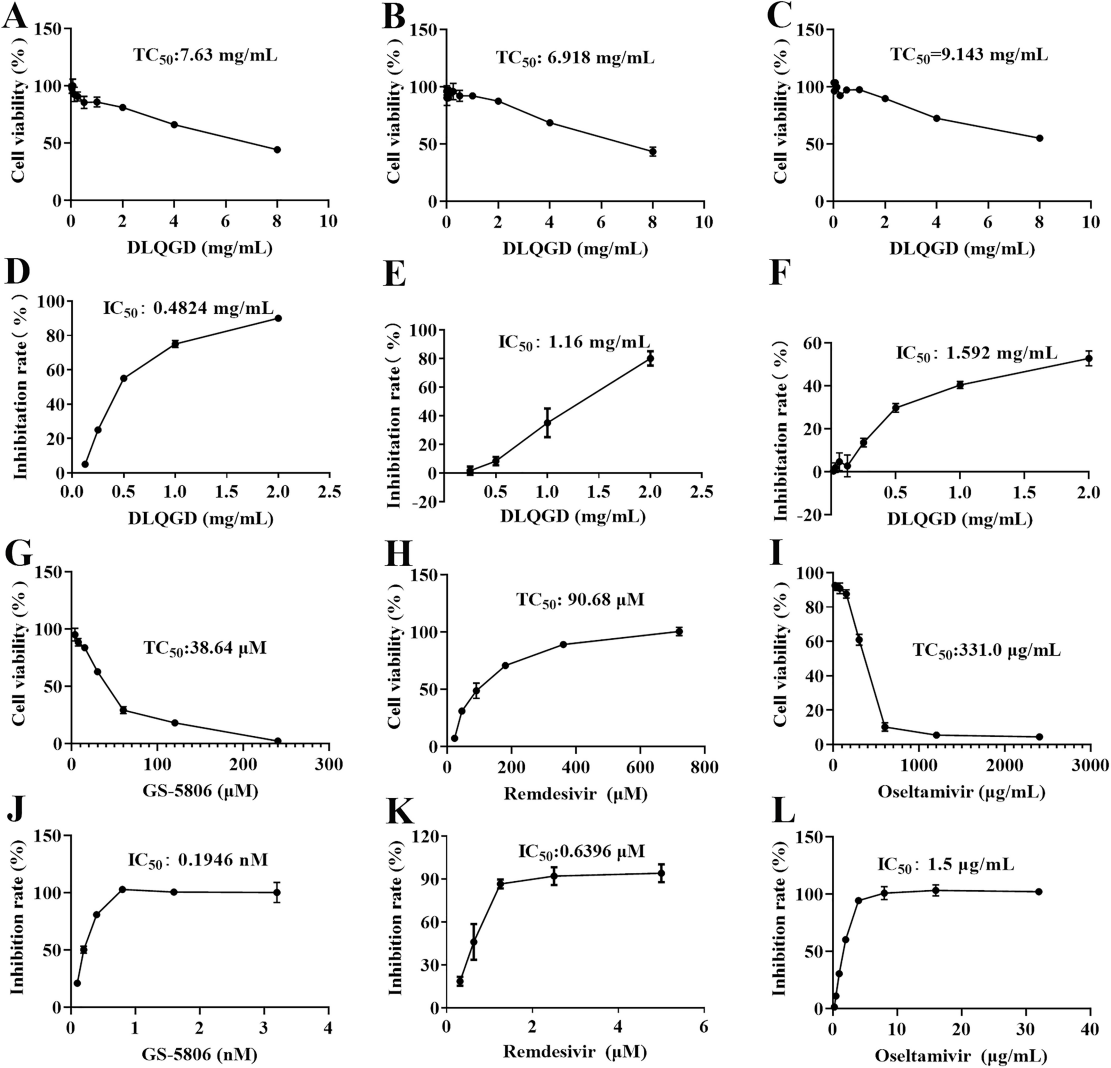
The antiviral effects of DLQGD against respiratory virus. **(A)** Cytotoxic effect of DLQGD on MDCK cells; **(B)** Cytotoxic effect of DLQGD on Vero E6 cells; **(C)** Cytotoxic effect of DLQGD on HEp-2 cells; **(D)** The antiviral effects of DLQGD against RSV; **(E)** The antiviral effects of DLQGD against SARS-CoV-2; **(F)** The antiviral effects of DLQGD against H3N2. **(G)** Cytotoxic effect of GS-5806 on HEp-2 cells; **(H)** Cytotoxic effect of Remdesivir on Vero E6 cells; **(I)** Cytotoxic effect of Oseltamivir on MDCK cells; **(J)** The antiviral effects of GS-5806 against RSV; **(K)** The antiviral effects of Remdesivir against SARS-CoV-2; **(L)** The antiviral effects of Oseltamivir against H3N2.

**FIGURE 8:**
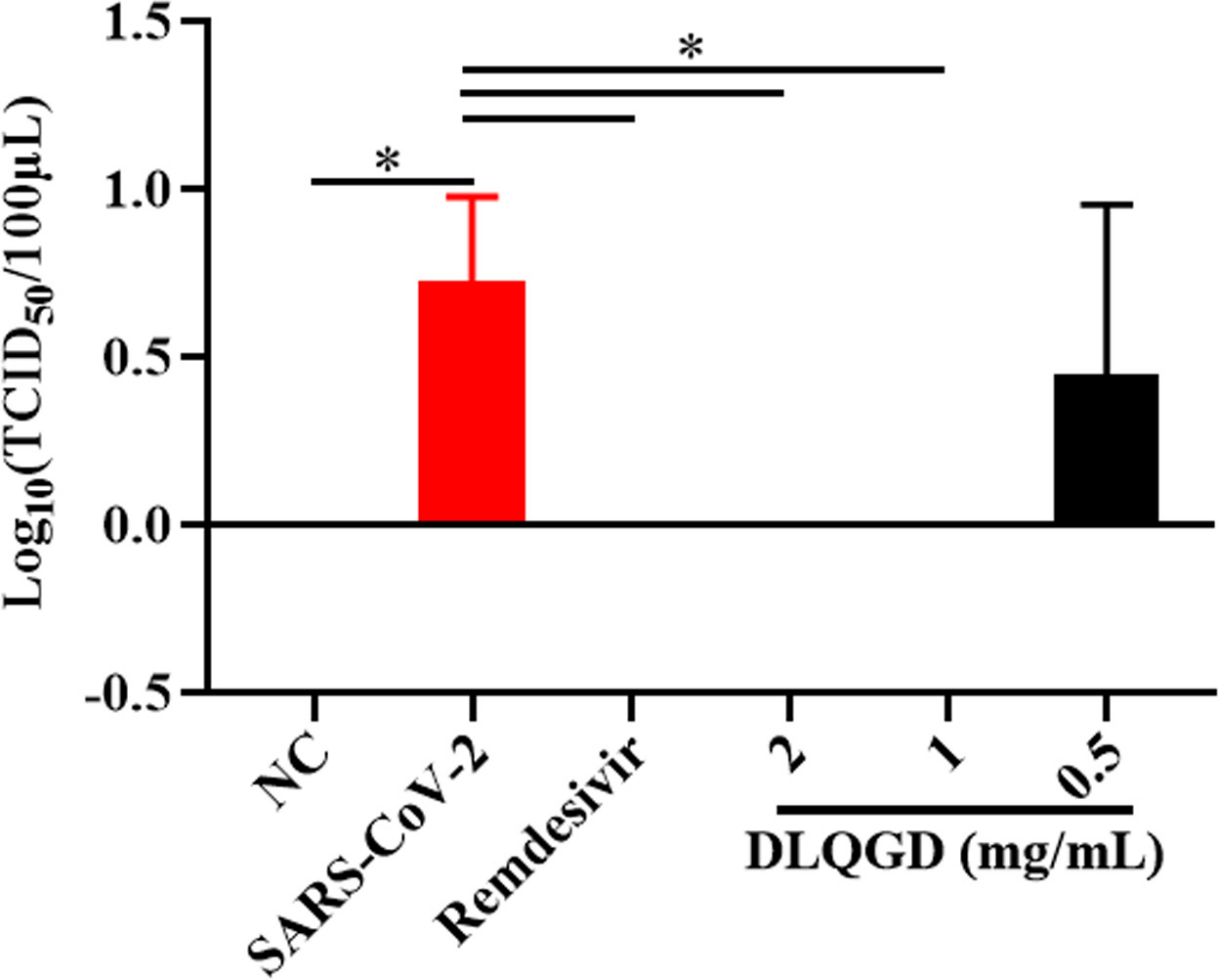
The inhibitory effects of DLQGD against SARS-CoV-2. Viral titer of collected cell culture supernatants in different group. The data were shown as mean ± SD and analyzed by one-way ANOVA Bonferroni or Dunnett’s multiple comparisons tests (*n* = 3). *p < 0.05. vs. SARS-CoV-2 group.

The antiviral effects of DLQGD on different subtypes of respiratory virus strains, including H3N2, SARS-CoV-2, and RSV, were determined by MTT assays or CPE assays. The median toxic concentration (TC_50_) of DLQGD in MDCK, Vero E6, and HEp-2 cells was 7.63, 6.918, and 9.143 mg/mL, respectively (Figures 7A–C). The 50% inhibitory concentration (IC_50_) of DLQGD against RSV, SARS-CoV-2, and H3N2 was 0.4824, 1.16, and 1.592 mg/mL (Figures 7D–F). The selectivity index (SI) of DLQGD against RSV, SARS-CoV-2, and H3N2 was 15.82, 5.96, and 5.74, respectively (Table 4). The IC_50_ of GS-5806, remdesivir and oseltamivir against RSV, SARS-CoV-2 and H3N2 was 0.1946 nM, 0.6396 μM, and 1.5 μg/mL (Figures 7G–L). These results indicated that DLQGD protect cells from cell death caused by RSV, SARS-CoV-2, and H3N2. In addition, cell culture supernatants were collected to determine the progeny virus production. Results showed that DLQGD (2 and 1 mg/mL) significantly inhibited the viral titer of cell culture supernatants during SARS-CoV-2 infection (Figure 8).

**TABLE 4:** Anti-viral effects of DLQGD *in vitro.*

Virus strains	DLQGD (mg/mL)			GS-5806 (μ M)			Oseltamivir (μ g/mL)			Remdesivir (μ M)		
	TC_50_	IC_50_	SI	TC_50_	IC_50_	SI	TC_50_	IC_50_	SI	TC_50_	IC_50_	SI
RSV	7.63	0.48	15.82	38.64	0.00019	> 10^3^	/	/	/	/	/	/
SARS-CoV-2	6.92	1.16	5.96	/	/	/	/	/	/	90.7	0.64	> 10^2^
A/Aichi/68	9.14	1.59	5.74	/	/	/	331	1.5	> 10^2^	/	/	/

### 3.7 Inhibitory effects of DLQGD on RSV-induced inflammatory response *in vitro*

**FIGURE 9:**
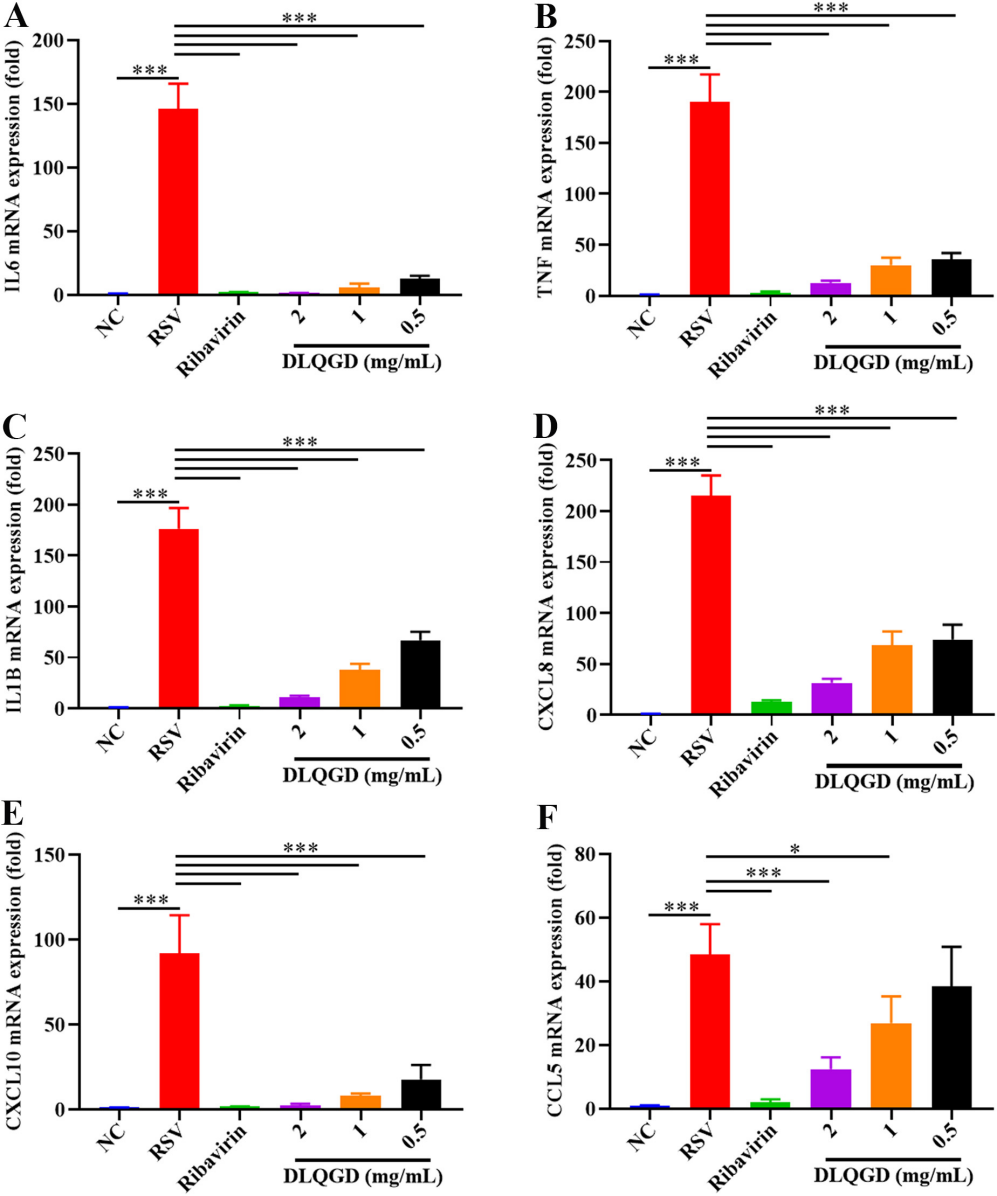
DLQGD inhibited RSV-induced inflammatory response in HEp-2 cells. **(A)** The mRNA expression of IL6 in HEp-2 cells. **(B)** The mRNA expression of TNF in HEp-2 cells. **(C)** The mRNA expression of IL1B in HEp-2 cells. **(D)** The mRNA expression of CXCL8 in HEp-2 cells. **(E)** The mRNA expression of CXCL10 in HEp-2 cells. **(F)** The mRNA expression of CCL5 in HEp-2 cells. The data were shown as mean ± SD and analyzed by one-way ANOVA Bonferroni or Dunnett’s multiple comparisons tests (*n* = 3). *p < 0.05 or ***p < 0.001. vs. RSV group.

The impact of DLQGD on the suppression of RSV-induced inflammatory responses was assessed *in vitro*. As shown in Figure 9, the mRNA expression levels of IL6, TNF, IL1B, CXCL8, CXCL10, and CCL5 were significantly upregulated in the RSV group compared with the normal control (NC) group (*p* < 0.001) (Figures 9A–F). DLQGD (1, 0.5, and 0.25 mg/mL) could significantly inhibit the mRNA expression levels of IL6, TNF, IL1B, CXCL8, and CXCL10 (*p* < 0.001) (Figures 9A–E). In addition, DLQGD (1, 0.5 and 0.25 mg/mL) could significantly suppress the mRNA expression of CCL5 (Figure 9F) (*p* < 0.001 or *p* < 0.05). These results indicated that DLQGD could inhibit RSV-induced inflammatory response.

### 3.8 Inhibitory effects of DLQGD on SARS-CoV-2-induced inflammatory response *in vitro*

**FIGURE 10:**
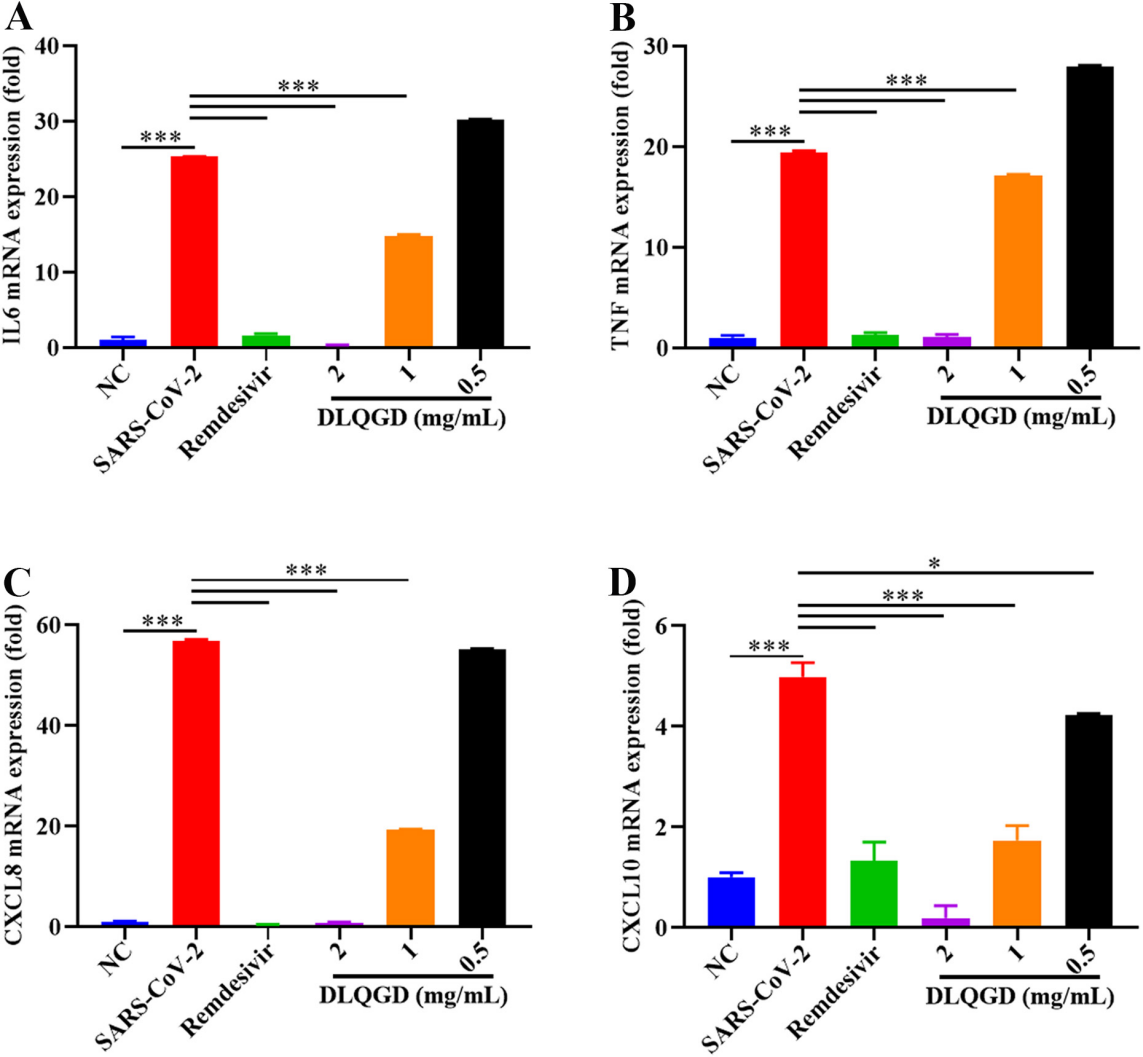
DLQGD inhibited SARS-CoV-2 induced inflammatory response *in vitro*. **(A)** The mRNA expression of IL6 in different groups. **(B)** The mRNA expression of TNF in different groups. **(C)** The mRNA expression of CXCL8 in different groups. **(D)** The mRNA expression of CXCL10 in different groups. The data were shown as mean ± SD and analyzed by one-way ANOVA Bonferroni or Dunnett’s multiple comparisons tests (*n* = 3). *p < 0.05 or ***p < 0.001. vs. SARS-CoV-2 group.

SARS-CoV-2 infection can trigger the innate immune system and induce an uncontrolled inflammatory response, which is associated with poor outcomes and high mortality (27). The effects of DLQGD on inhibiting SARS-CoV-2-induced inflammatory response were evaluated *in vitro*. As shown in Figure 10, SARS-CoV-2 infection induced high levels of IL6, TNF, CXCL8, and CXCL10 compared with the NC group. Notably, DLQGD significantly inhibited the mRNA expression of IL6, TNF, CXCL8, and CXCL10. These results indicated that DLQGD can inhibit SARS-CoV-2-induced overactivated inflammatory response.

### 3.9 Inhibitory effects of DLQGD on SARS-CoV-2-induced activated signaling pathways *in vitro*

**FIGURE 11:**
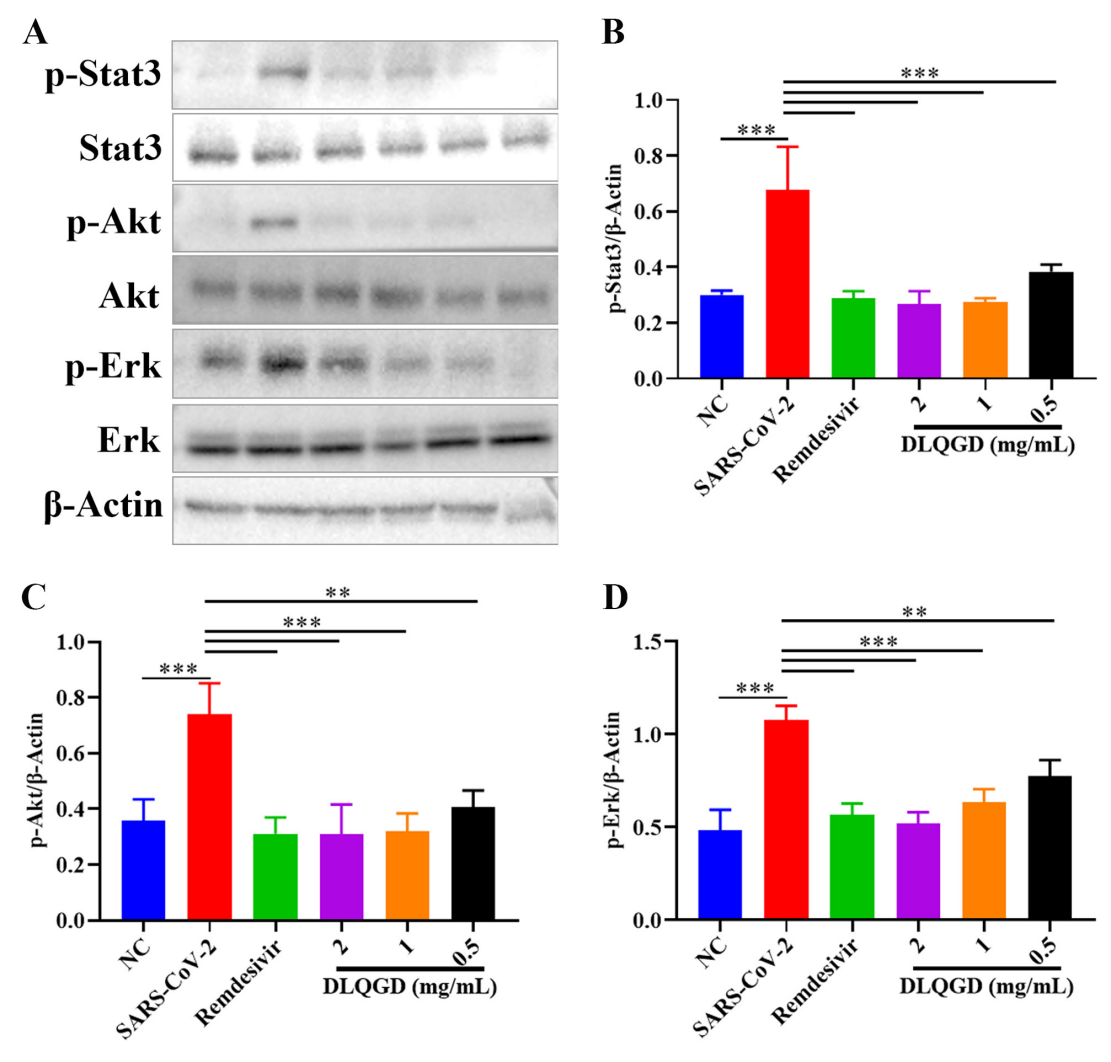
DLQGD inhibited SARS-CoV-2-induced activated signaling pathways *in vitro*. **(A)** The expression of p-Stat3, Stat3, p-Akt, Akt, p-Erk1/2, Erk1/2, and β-Actin in different groups during SARS-CoV-2 infection *in vitro*. **(B–D)** The relative expression of p-Stat3, p-Akt, and p-Erk1/2 analyzed by Image J. The data were shown as mean ± SD and analyzed by one-way ANOVA Bonferroni or Dunnett’s multiple comparisons tests (*n* = 3). **p < 0.01 or ***p < 0.001. vs. SARS-CoV-2 group.

KEGG enrichment analysis indicated that DLQGD could regulate PI3K-Akt signaling pathway, Stat3 signaling pathway and MAPK signaling pathway in viral pneumonia, which are associated with virus-induced inflammatory response. SARS-CoV-2 induced increased phosphorylation of Stat3, Akt and Erk1/2, which could be reduced by DLQGD (Figures 11A–D). These results showed that DLQGD alleviated the overactivated inflammatory response by inhibiting PI3K-Akt signaling pathway, Stat3 signaling pathway and ERK MAPK signaling pathways.

## 4 Discussion

Respiratory viral infections pose a significant threat to public health. Virus-induced overactivated inflammatory response can drive the formation of cytokine storm. The pathogenesis of a cytokine storm during respiratory virus infection is a severe dysregulation of the innate immune response. Upon invading the respiratory tract, the virus infects epithelial cells, replicating extensively and activating the cascade of signaling pathway. This process initiates the first wave of inflammatory response and recruits various innate cells, such as neutrophils and macrophages, into the lungs. In addition, in an attempt to control the rampant viral replication, these innate immune cells become hyperactivated and initiate an exaggerated release of a vast array of pro-inflammatory cytokines and chemokines, such as TNF-α, CXCL8, IL-6, IL-1β, and IFN-γ. This inflammatory cascade amplifies uncontrollably, leading to a systemic surge of these inflammatory mediators. The formation of cytokine storm recruits an overwhelming number of immune cells into the lungs, leading to widespread epidermal-endothelial damage and increased vascular permeability. The consequence is severe tissue pathology, pneumonia, ARDS, and often multi-organ failure, which are the primary causes of mortality in severe infection. Effective strategies are needed to control virus-induced cytokine storm. A previous study has found that DLQGD possesses anti-inflammatory effects against HCoV-229E. Despite ongoing research, a comprehensive understanding of the potential mechanisms underlying DLQGD’s therapeutic effects in viral pneumonia remains elusive. Consequently, this study utilized an integrated methodology, incorporating network pharmacology, molecular docking, and preliminary *in vitro* experiments, to systematically elucidate the multi-component, multi-target, and multi-pathway molecular mechanisms of DLQGD in the treatment of viral pneumonia.

In this study, we first identified the chemical components of DLQGD using HPLC-Q-TOF MS, providing insight into the potential anti-inflammatory effects of DLQGD. Some of these components, such as isorhamnetin, hesperidin, quercetin, sinensetin and rosmarinic acid, can inhibit influenza virus-induced inflammation (28–32). Luteolin, acteoside and tangeretin can alleviate RSV-induced overactivated inflammatory response (33–35). Other ingredients, including nobiletin, glycyrrhizic acid, isoliquiritin and naringin, have also demonstrated anti-inflammatory effects (36–39). These results suggest that DLQGD contains multiple active compounds that may contribute to its inhibitory effects on virus-induced inflammatory response.

Network pharmacology analysis identified a wide array of targets regulated by active pharmaceutical ingredients of DLQGD. KEGG enrichment analysis of these targets revealed significant associations with key KEGG signaling pathways, particularly the PI3K-Akt signaling pathways. Through the construction of a comprehensive PPI network, we identified six core targets, including STAT3, AKT1, PIK3CA, PIK3R1, JUN, and MAPK1, which are likely central to the mechanism of action of DLQGD in viral pneumonia. Among them, AKT1, PIK3CA, and PIK3R1 are key targets in the PI3K-Akt signaling pathway. The PI3K-Akt signaling pathway serves as a crucial modulator of both pro-inflammatory responses. This pathway is activated by various stimuli, including pathogen recognition and cytokine signals, and subsequently influences key inflammatory processes such as NF-κB activation, NLRP3 inflammasome assembly, and the production of cytokines like IL-1β, IL-6, and TNF-α. In the setting of viral pneumonia, the PI3K-Akt signaling pathway, Stat3 signaling pathway and MAPK pathway are frequently activated by viruses such as influenza, RSV and SARS-CoV-2, promoting a hyperinflammatory state that contributes to the cytokine storm and acute lung injury. Targeting these pathways have emerged as a promising therapeutic strategy. Through the construction of a target–active ingredient network, six key components, including tangeretin, isorhamnetin, isolicoflavonol, luteolin, sinensetin and acacetin, were identified. Subsequent molecular docking studies revealed favorable binding affinities between six principal components and core targets (STAT3, MAPK1, AKT1, PIK3CA, and PIK3R1). Western blot experiments also confirmed the inhibitory effects of DLQGD on phosphorylated Stat3, Akt and Erk. Therefore, DLQGD exhibits anti-inflammatory effects probably by inhibiting the activation of inflammatory signaling pathways such as the PI3K-Akt signaling pathway, Stat3 signaling pathway and MAPK pathway.

While this study provides a systematic network pharmacology analysis and preliminary *in vitro* validation of DLQGD’s anti-inflammatory mechanisms, it is important to acknowledge its limitations. The absence of *in vivo* experiments remains a key constraint, as the complex pathophysiology of viral pneumonia including immune cell recruitment and tissue damage, cannot be fully recapitulated in cell-based models. Therefore, the translational relevance of our findings warrants further investigation in a living organism. To address this, future studies should prioritize employing well-established animal models of viral pneumonia (e.g., RSV or SARS-CoV-2-infected mice) to evaluate the *in vivo* efficacy of DLQGD. In addition, future studies will be essential to comprehensively evaluate the anti-respiratory virus potential of the key active components such as tangeretin, isorhamnetin, isolicoflavonol, luteolin, sinensetin, and acacetin.

## 5 Conclusion

In conclusion, DLQGD confers an inhibitory effect on respiratory virus-induced inflammation. It is mainly attributed to the inhibition of the activation of the PI3K-Akt pathway, Stat3 signaling pathway and MAPK pathway. DLQGD may serve as a promising agent candidate for the treatment of respiratory virus infection.

## Data Availability

The original contributions presented in the study are included in the article/Supplementary material, further inquiries can be directed to the corresponding authors.

## Footnotes

1
https://pubchem.ncbi.nlm.nih.gov/

2
https://www.swisstargetprediction.ch/

3
https://www.uniprot.org

4
https://bioinfogp.cnb.csic.es/tools/venny/

5
https://cn.string-db.org/

6
https://davidbioinformatics.nih.gov/gene2gene_new.jsp

7
http://cao.labshare.cn/cb-dock/
